# Supplementary material for: The Novel A-Type Proanthocyanidin-Rich Phytocomplex SP4™ Acts as a Broad-Spectrum Antiviral Agent against Human Respiratory Viruses
Source: Int J Mol Sci. 2024 Jul 5;25(13):7370. doi: 10.3390/ijms25137370 (PMC11242173; doi:10.3390/ijms25137370)
Supplement: Supplementary file 1 [file ijms-25-07370-s001.zip › ijms-3053445-supplementary.pdf]

Supplementary Table S1. Identification of the phytochemicals of primary and secondary SP4™ fractions[1].

Chemical Name	*m/z*	MS/MS	Fraction 1	1A	1B	1 C	Fraction 2	Fraction 3	3A	3B	3C	3D	3E
Cyanidin 3-O-arabinoside	419	286.8	tr[2]	tr	tr	n.d.[3]	58.79 ± 1.73	n.d.	n.d.	n.d.	n.d.	n.d.	n.d.
Cyanidin 3-O-galactoside	449	286.9	tr	n.d.	tr	n.d.	54.67 ± 0.73	n.d.	n.d.	n.d.	n.d.	n.d.	n.d.
Cyanidin 3-O-glucoside	449	286.9	tr	tr	tr	n.d.	104.51 ± 3.93	n.d.	n.d.	n.d.	n.d.	n.d.	n.d.
Peonidin 3-O-galactoside	463	301	tr	tr	tr	n.d.	98.46 ± 1.36	n.d.	n.d.	n.d.	n.d.	n.d.	n.d.
Peonidin 3-O-glucoside	463	300.9	tr	tr	tr	n.d.	139.30 ± 2.35	n.d.	n.d.	n.d.	n.d.	n.d.	n.d.
Peonidin 3-O-arabinoside	473	455.1, 427.0, 300.9	tr	tr	tr	n.d.	100.77 ± 3.05	n.d.	n.d.	n.d.	n.d.	n.d.	n.d.
PAC-A1	575	497.7; 448.7; 410.8; 288.7	tr	n.d.	tr	n.d.	tr	29.28 ± 0.88	28.35 ± 1.13	1.25 ± 0.03	61.02 ± 1.58	15.91 ± 0.50	n.d.
PAC-A2	575	497.7; 448.7; 410.8; 288.7	tr	n.d.	tr	n.d.	tr	56.07 ± 1.09	35.74 ± 0.50	1.45 ± 0.02	48.61 ± 1.81	13.50 ± 0.23	n.d.
PAC-A4	575	497.7; 448.7; 410.8; 288.7	tr	n.d.	tr	n.d.	tr	31.75 ± 0.62	33.95 ± 0.71	1.15 ± 0.04	59.64 ± 0.77	13.86 ± 0.19	n.d.
PAC-B (4-8)	577	450.8; 424.8; 406.8; 289	tr	n.d.	tr	n.d.	tr	51.38 ± 0.81	6.01 ± 0.09	n.d.	5.80 ± 0.18	1.09 ± 0.04	tr
PAC-B3	577	450.8; 424.8; 406.8; 289	tr	n.d.	tr	n.d.	tr	14.82 ± 0.28	6.54 ± 0.26	n.d.	6.82 ± 0.20	0.92 ± 0.01	tr
PAC-B2	577	450.8; 424.8; 406.8; 289	tr	n.d.	tr	n.d.	tr	35.39 ± 1.01	5.30 ± 0.06	n.d.	6.01 ± 0.07	1.02 ± 0.02	tr
Unk.1 PAC-B*	577	450.8; 424.8; 406.8; 289	tr	n.d.	tr	n.d.	tr	51.38 ± 2.02	5.64 ± 0.11	n.d.	5.26 ± 0.20	1.26 ± 0.01	tr
Unk 2 PAC-B*	577	450.8; 424.8; 406.8; 289	tr	n.d.	tr	n.d.	tr	59.12 ± 1.91	4.63 ± 0.17	n.d.	6.37 ± 0.17	0.99 ± 0.03	tr
Cyanidin	287	258.9; 230.9	tr	n.d.	tr	tr	30.05 ± 0.36	n.d.	n.d.	n.d.	n.d.	n.d.	n.d.

Peonidin	301	240.1	0.80 ± 0.01	n.d.	tr	1.00 ± 0.03	37.52 ± 1.26	n.d.	n.d.	n.d.	n.d.	n.d.	n.d.
PAC-A3	575	497.7; 448.7; 410.8; 288.7	1.04 ± 0.02	tr	tr	n.d.	tr	25.32 ± 0.99	25.67 ± 0.66	1.35 ± 0.04	47.41 ± 1.55	15.32 ± 0.61	n.d.
PAC-B1	577	450.8; 424.8; 406.8; 289	1.63 ± 0.05	tr	0.54 ± 0.02	n.d.	tr	37.17 ± 0.83	4.81 ± 0.09	n.d.	7.49 ± 0.13	0.93 ± 0.03	tr
Unk 3 PAC-B*	577	450.8; 424.8; 406.8; 289	1.22 ± 0.02	tr	tr	n.d.	tr	63.73 ± 1.34	5.27 ± 0.07	n.d.	6.14 ± 0.14	1.20 ± 0.04	tr
Unk 4 PAC-B*	577	450.8; 424.8; 406.8; 289	1.07 ± 0.04	0.56 ± 0.02	tr	n.d.	tr	51.38 ± 1.83	4.75 ± 0.10	n.d.	7.41 ± 0.22	1.20 ± 0.02	tr
Dihydroquercetin-3-O-glucoside	465	436.7; 302.7	18.71 ± 0.42	252.12 ± 6.03	0.75 ± 0.01	tr	8.35 ± 0.22	2.67 ± 0.10	n.d.	n.d.	n.d.	n.d.	n.d.
Dihydroquercetin-7-O-diglucoside	611	574.7; 422.7	18.96 ± 0.37	107.76 ± 1.99	5.80 ± 0.07	n.d.	tr	tr	n.d.	n.d.	n.d.	n.d.	n.d.
Dihydromyricetin 3,4′-O-diglucoside	643	596.6; 490.6; 318.7	69.16 ± 1.54	155.02 ± 4.14	0.28 ± 0.01	tr	1.42 ± 0.04	n.d.	n.d.	n.d.	n.d.	n.d.	n.d.
Quercetin 3-O-sambubioside	597	548.9; 301.0	73.60 ± 2.24	97.75 ± 3.15	tr	n.d.	tr	n.d.	n.d.	n.d.	n.d.	n.d.	n.d.
Catechin	289	270.8	75.78 ± 2.55	4.09 ± 0.08	94.40 ± 3.62	tr	tr	15.40 ± 0.21	tr	n.d.	3.30 ± 0.04	n.d.	n.d.
Quercetin 3-O-rutinoside	609	342.7; 300.7	44.42 ± 0.90	5.22 ± 0.14	183.21 ± 3.86	tr	tr	tr	n.d.	n.d.	n.d.	n.d.	n.d.
Epicatechin	289	270.8	12.83 ± 0.20	8.20 ± 0.09	62.37 ± 2.17	0.84 ± 0.01	tr	7.03 ± 0.21	tr	n.d.	2.78 ± 0.03	n.d.	n.d.
Quercetin 3-O-galactoside	463	416.7; 300.7	35.99 ± 0.93	21.58 ± 0.25	117.48 ± 2.91	0.85 ± 0.03	tr	0.72 ± 0.01	n.d.	n.d.	n.d.	n.d.	n.d.
Quercetin 3-O-glucuronide	477	300.7	46.44 ± 1.68	4.78 ± 0.13	58.34 ± 1.68	n.d.	tr	tr	n.d.	n.d.	n.d.	n.d.	n.d.
3′-Methylquercetin 3-O-galactoside	623	314.7; 299.7; 270.7	36.99 ± 1.16	0.87 ± 0.01	81.67 ± 2.15	tr	n.d.	tr	n.d.	n.d.	n.d.	n.d.	n.d.
Myricetin 3-O-rutinoside	625	317.1	36.75 ± 0.77	0.86 ± 0.01	126.01 ± 1.29	tr	n.d.	n.d.	n.d.	n.d.	n.d.	n.d.	n.d.
3′-Methylquercetin 3-O-glucoside	477	356.7; 314.7	33.38 ± 0.68	n.d.	tr	74.49 ± 2.55	tr	tr	n.d.	n.d.	n.d.	n.d.	n.d.
Quercetin 3-O-glucoside	463	416.7; 300.7	29.06 ± 0.46	n.d.	tr	85.78 ± 1.04	tr	0.72 ± 0.02	n.d.	n.d.	n.d.	n.d.	n.d.
Quercetin	301	256.8	75.11 ± 1.98	n.d.	tr	93.80 ± 3.39	tr	1.25 ± 0.01	n.d.	n.d.	n.d.	n.d.	n.d.
Dihydroquercetin	303	284.7	14.14 ± 0.54	n.d.	tr	56.71 ± 1.29	tr	n.d.	n.d.	n.d.	n.d.	n.d.	n.d.

Dihydromyricetin	319	295.4	11.81 ± 0.20	n.d.	tr	97.78 ± 3.66	tr	n.d.	n.d.	n.d.	n.d.	n.d.	n.d.
Myricetin	317	297.9; 270.7	18.67 ± 0.44	n.d.	tr	106.42 ± 2.04	6.44 ± 0.12	0.50 ± 0.01	n.d.	n.d.	n.d.	n.d.	n.d.
3′-Methylquercetin	315	297	98.68 ± 1.99	n.d.	tr	93.29 ± 1.17	2.45 ± 0.09	1.31 ± 0.03	n.d.	n.d.	n.d.	n.d.	n.d.
A-type PAC trimer	861	575; 289	n.d.	n.d.	n.d.	n.d.	n.d.	2.28 ± 0.07	2.50 ± 0.03	n.d.	3.09 ± 0.07	4.35 ± 0.06	n.d.
AB-type PAC trimer	863	575; 289	n.d.	n.d.	n.d.	n.d.	n.d.	35.12 ± 0.76	62.67 ± 2.23	2.30 ± 0.09	80.60 ± 1.42	7.57 ± 0.25	n.d.
B-type PAC trimer	865	577; 289	n.d.	n.d.	n.d.	n.d.	n.d.	11.71 ± 0.30	24.98 ± 0.59	tr	34.07 ± 1.2	1.18 ± 0.03	n.d.
B-type PAC tetramer	1147	861; 289	n.d.	n.d.	n.d.	n.d.	n.d.	5.77 ± 0.07	n.d.	n.d.	1.30 ± 0.05	0.80 ± 0.02	n.d.
A2B4-type PAC hexamer	1149	861; 575; 289	n.d.	n.d.	n.d.	n.d.	n.d.	9.90 ± 0.11	21.34 ± 0.55	0.95 ± 0.04	16.42 ± 0.64	10.96 ± 0.20	tr
A1B4-type PAC pentamer	1151	861; 575; 289	n.d.	n.d.	n.d.	n.d.	n.d.	12.39 ± 0.38	19.49 ± 0.25	0.63 ± 0.02	16.68 ± 0.39	8.53 ± 0.20	n.d.
B4-type PAC tetramer	1153	863; 575; 577; 289	n.d.	n.d.	n.d.	n.d.	n.d.	3.16 ± 0.06	11.26 ± 0.41	n.d.	1.013 ± 0.04	1.31 ± 0.03	n.d.
3A1B-type PAC tetramer	1435	1153; 577; 289	n.d.	n.d.	n.d.	n.d.	n.d.	7.52 ± 0.24	134.08 ± 1.54	n.d.	94.28 ± 1.16	69.88 ± 1.75	n.d.
A2B2-type PAC tetramer	1437	1147; 577; 289	n.d.	n.d.	n.d.	n.d.	n.d.	29.46 ± 0.46	57.27 ± 1.07	16.44 ± 0.47	55.02 ± 2.19	25.26 ± 0.78	n.d.
A1B5-type PAC hexamer	1439	1149; 861; 575; 289	n.d.	n.d.	n.d.	n.d.	n.d.	38.47 ± 0.60	80.94 ± 1.51	17.25 ± 0.50	68.21 ± 2.71	119.95 ± 3.69	2.12 ± 0.08
B5-Type PAC pentamer	1441	1149; 861; 575; 289	n.d.	n.d.	n.d.	n.d.	n.d.	12.27 ± 0.19	265.60 ± 4.97	3.75 ± 0.11	203.48 ± 8.09	37.56 ± 1.15	6.19 ± 0.22
3A3B-Type PAC hexamer	1724	1435; 1149; 861; 575; 289	n.d.	n.d.	n.d.	n.d.	n.d.	14.02 ± 0.22	27.73 ± 0.52	n.d.	21.34 ± 0.85	14.89 ± 0.46	n.d.
A2B6-type PAC hexamer	1726	863; 575; 577; 289	n.d.	n.d.	n.d.	n.d.	n.d.	12.20 ± 0.19	25.91 ± 0.49	tr	21.13 ± 0.84	4.59 ± 0.14	n.d.
A4B6-type PAC hexamer	1728	1149; 861; 575; 289	n.d.	n.d.	n.d.	n.d.	n.d.	3.74 ± 0.06	5.64 ± 0.11	n.d.	7.54 ± 0.30	1.12 ± 0.03	n.d.
B-type PAC hexamer	1730	1153; 577; 289	n.d.	n.d.	n.d.	n.d.	n.d.	7.13 ± 0.11	tr	n.d.	n.d.	n.d.	n.d.

[1]Values are expressed as mg/g (± SD).
[2]tr = compounds below 0.5 mg/g.
[3]n.d. = not detectable.
*For these PAC-Bs it was not possible to determine the exact structure that remains unknown, but only the molecular mass was determined.

PAC-A1 **PAC-A2**

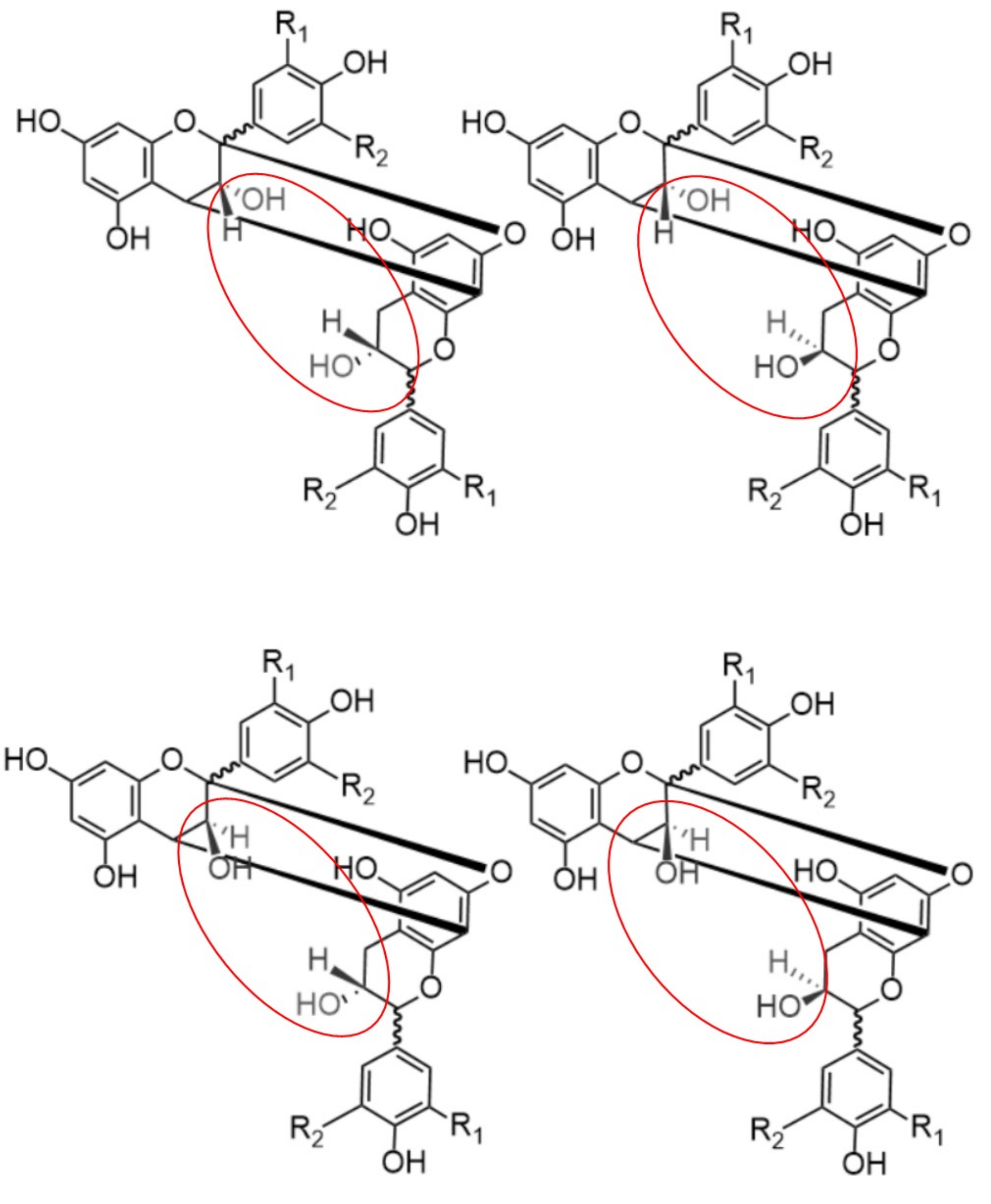

PAC-A3 **PAC-A4**

Supplementary Figure S1. Chemical structures of dimeric A-type proanthocyanidins (PAC-As) present in SP4™ and endowed with antiviral activity. The wedged and dashed lines indicate chemical bonds directed above or below the plane of the figure, respectively. The red circles highlight the functional groups whose different stereoisomerism results in four distinctive PACs-As dimers (A1-4).
